# Pediatric Sigmoid Volvulus Presenting As Acute Intestinal Obstruction in a Previously Healthy 14-Year-Old Adolescent: A Case Report

**DOI:** 10.7759/cureus.100535

**Published:** 2025-12-31

**Authors:** Nehemia I Kassa, Efeson T Malore, Nigussie Mohammed, Yosef Alemayehu, Kaleab Zereai

**Affiliations:** 1 Surgery, Myungsung Christian Medical Center, Addis Ababa, ETH; 2 General Surgery, Myungsung Christian Medical Center, Addis Ababa, ETH; 3 Internal Medicine, Myungsung Christian Medical Center, Addis Ababa, ETH

**Keywords:** acute abdomen, case report, pediatric bowel obstruction, pediatric surgery, sigmoid volvulus

## Abstract

Sigmoid volvulus is the torsion of the sigmoid colon around its mesenteric axis. It is a rare cause of intestinal obstruction in children and may be overlooked due to its nonspecific presentation. We describe the case of a previously healthy 14-year-old female who presented with acute abdominal pain, progressive distension, and prolonged obstipation, with imaging demonstrating a markedly dilated sigmoid colon and the characteristic “coffee-bean" sign consistent with sigmoid volvulus. She underwent emergency laparotomy, and intraoperative evaluation revealed a redundant, twisted sigmoid colon with preserved viability. She underwent successful detorsion, resection of the redundant sigmoid colon, and primary anastomosis. This case underscores the importance of maintaining a high index of suspicion for sigmoid volvulus in children presenting with acute intestinal obstruction, even in the absence of identifiable risk factors, as well as the need for prompt diagnosis using imaging and timely surgical intervention to prevent bowel ischemia and life-threatening complications.

## Introduction

Sigmoid volvulus refers to torsion of the sigmoid colon around its mesenteric axis, resulting in compromised intestinal lumen and vascular supply [1]. It is a well-recognized cause of large-bowel obstruction in adults, particularly in regions where diets are rich in fiber [2]. However, sigmoid volvulus is uncommon in the pediatric population, accounting for only 0.4-1% of childhood intestinal obstruction cases [3], and its rarity often contributes to delayed diagnosis and higher complication rates.

The etiology in children is multifactorial and may involve congenital anatomic predispositions such as redundant sigmoid colon, narrow mesenteric base, or elongated mesentery, as well as motility disorders including Hirschsprung's disease, chronic constipation, and neuromuscular conditions [4]. In many cases, no clear predisposing factor is identified [3].

Clinically, pediatric sigmoid volvulus may present with abdominal pain, distention, constipation, bilious vomiting, and progressive bowel obstruction [5]. However, symptoms often overlap with common pediatric gastrointestinal disorders, making early recognition challenging. Radiographs may demonstrate classic findings such as the “coffee-bean” sign. Prompt diagnosis and timely intervention are crucial to prevent bowel ischemia, necrosis, and perforation [4,6]. This case highlights the rare occurrence of sigmoid volvulus in a previously healthy adolescent and underscores the importance of maintaining a high index of suspicion for volvulus in children presenting with acute intestinal obstruction.

## Case presentation

A 14-year-old previously healthy female presented to the emergency department with diffuse lower abdominal pain of 12 hours' duration. The pain was intermittent, cramping in nature, and rated 5/10 in severity. It was associated with progressive abdominal distension and cessation of feces and flatus for the past 48 hours. She also experienced anorexia, nausea, and a single episode of non-bilious vomiting. She had no history of similar symptoms. She denied fever, chills, chronic constipation, recurrent abdominal pain, alternating diarrhea and constipation, joint pain, visual symptoms, or back pain. She had no history of previous surgery, weight loss, bloody stools, or a family history of colonic or rectal cancer. Her diet was balanced, consisting of injera, meat, grains, vegetables, and fruits. She had no known chronic medical illnesses and was not taking any medications.

On physical examination, the patient appeared acutely ill and in mild distress due to pain. Vital signs were stable: blood pressure 120/80 mmHg, heart rate 94 beats per minute, respiratory rate 18 breaths per minute, temperature 36.0°C, and oxygen saturation 96% on room air. Her anthropometric profile was age-appropriate, with a height of 1.75 m, a weight of 65 kg, and a BMI of 21 kg/m^2^. Abdominal examination revealed mild distension with normal respiratory movement. Bowel sounds were hyperactive, and the abdomen was grossly hyper-tympanic on percussion. On palpation, the abdomen was soft with mild diffuse lower abdominal tenderness, more pronounced in the left lower quadrant. There was no rigidity or rebound tenderness, and all appendiceal signs were negative. Digital rectal examination revealed no external hemorrhoids, fissures, or other abnormalities. Rectal tone was normal, and the rectal vault was empty with no palpable masses or blood on the examining finger. The remainder of the physical examination was unremarkable.

Laboratory investigations demonstrated mildly elevated WBC count (11×10^9^/L) with normal hemoglobin, platelet count, C-reactive protein (CRP), and renal function test levels (Table 1).

**Table 1:** Patient’s preoperative laboratory test results. Reference values were obtained from standard laboratory reference ranges [7]. WBC: white blood cell; BUN: blood urea nitrogen

Investigation	Patient’s Result	Reference Values
WBC count	11,000/µL	5,000-10,000/µL
Hemoglobin	14.7g/dL	13-17g/dL
Platelet count	250,000/µL	150-400,000/µL
C-reactive protein	5mg/L	<10mg/L
Kidney profile - BUN	10mg/dL	7-20mg/dL
Kidney profile - creatinine	0.8mg/dL	0.7-1.3mg/dL

**Figure 1:**
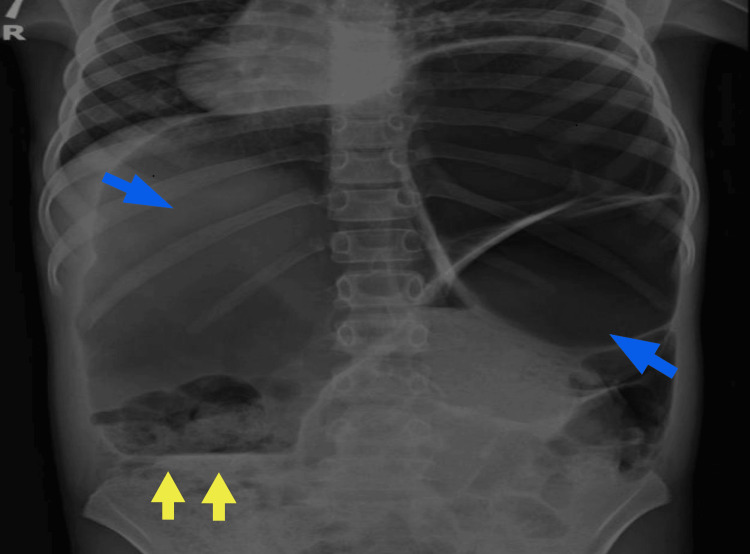
Abdominal radiograph demonstrating dilated large-bowel loops with peripheral air-fluid levels (yellow arrows) and the classic “coffee-bean” sign (blue arrows).

Plain abdominal and chest radiography (Figure 1) revealed a markedly dilated sigmoid colon with the classic “coffee-bean" sign, indicative of large bowel obstruction secondary to sigmoid volvulus. There was no free air in the peritoneum, suggestive of perforation.

**Figure 2:**
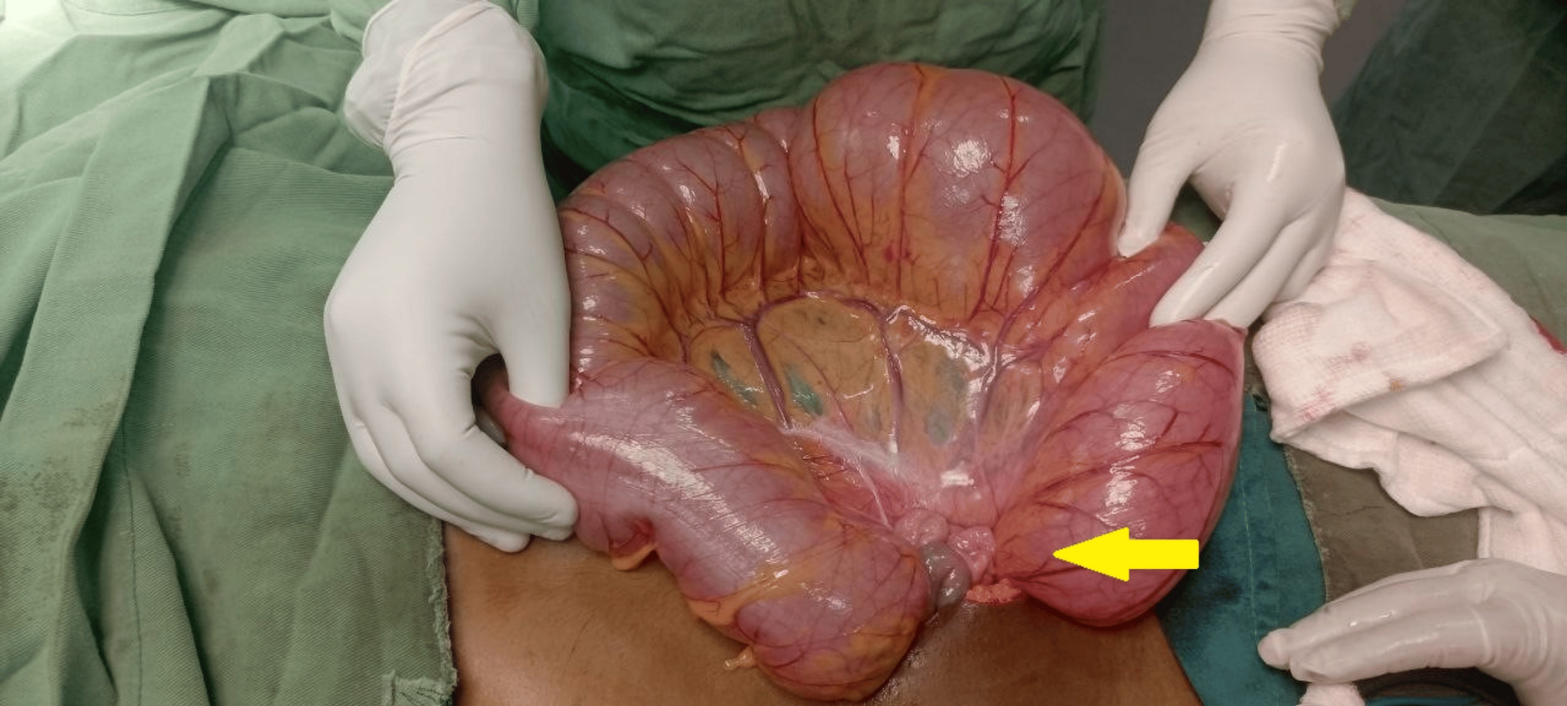
Intraoperative image showing a redundant sigmoid colon twisted around its mesentery (yellow arrow).

**Figure 3:**
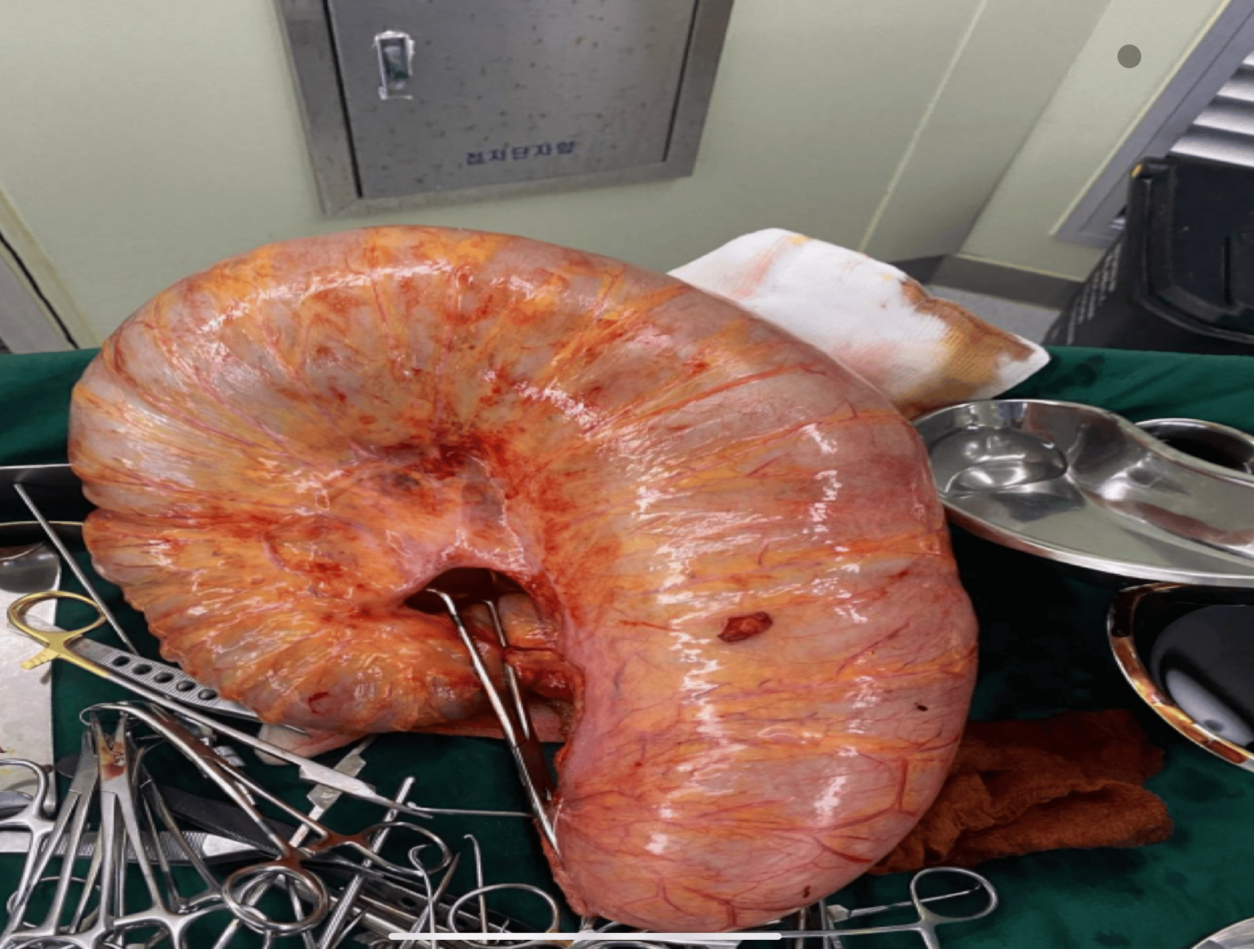
Resected specimen demonstrating a redundant sigmoid colon.

Based on the assessment of an acute abdomen secondary to large bowel obstruction due to sigmoid volvulus, the patient was resuscitated with intravenous fluids and started on broad-spectrum antibiotics (ceftriaxone and metronidazole). She was then prepared for emergency laparotomy, which was performed within two hours of presentation. Intra-operative findings demonstrated a redundant sigmoid colon twisted on its mesentery (Figure 2), with preserved vascularity and no evidence of gangrene or perforation. The volvulus was reduced, and segmental resection of the redundant sigmoid colon was performed (Figure 3) with primary end-to-end anastomosis. The resected bowel was sent for histological analysis, which revealed hypertrophy of the mucosa, muscularis propria, and enteric nerve plexuses, along with fibrosis in the mesentery and submucosa - findings consistent with volvulus-related changes.

The postoperative course was uncomplicated. The patient resumed bowel function, tolerated diet advancement, and was discharged on postoperative day 5 with scheduled follow-up. At her two-week outpatient review, she remained asymptomatic and clinically stable.

## Discussion

Sigmoid volvulus in children remains a diagnostic and therapeutic challenge due to its rarity and nonspecific presentation [3]. Unlike adults, where dietary and anatomic factors predominate, pediatric cases are often associated with congenital malrotation, redundant colon, Hirschsprung's disease, motility disorders, or chronic constipation [4]. Yet, many patients, including the one in this report, may present without identifiable risk factors [3]. This hugely underscores the importance of clinical vigilance.

Radiographic findings, particularly the “coffee-bean" sign, are suggestive but not pathognomonic [6]. Contrast enema can be both diagnostic and therapeutic, showing the “bird’s-beak" sign, and is recommended in stable children without peritonitis or ischemia [8]. Non-operative detorsion, using a contrast enema or flexible sigmoidoscopy, may relieve the obstruction and temporarily decompress the bowel [5,6]. However, recurrence is common, reported in up to 30-60% of cases [8,9], and delayed surgery increases the risk of ischemia and perforation. Therefore, non-operative management is generally a bridge to definitive surgery, with early elective resection recommended in children with redundant sigmoid colon [3,9]. In our case, the decision for surgery was guided by the patient’s severe and progressive symptoms and the associated risk of bowel ischemia, prompting immediate operative intervention to reduce the likelihood of recurrence and other complications.

Surgical intervention remains the definitive treatment. When the bowel is viable, options include detorsion with or without mesosigmoidoplasty, or resection with primary anastomosis [3,9]. Resection is often preferred in cases with redundant colon to minimize recurrence [9]. If gangrene is present, resection with temporary stoma (Hartmann procedure) may be required [8].

Pediatric sigmoid volvulus carries a significant risk of bowel ischemia, perforation, sepsis, and mortality, particularly when diagnosis is delayed [4,6]. Early recognition and appropriate surgical decision-making are crucial. This case reinforces that even previously healthy adolescents without significant risk factors can develop sigmoid volvulus and benefit from timely operative intervention to avoid life-threatening complications.

## Conclusions

Sigmoid volvulus is a rare but important cause of acute intestinal obstruction in the pediatric population. Its non-specific presentation can delay diagnosis, increasing the risk of bowel ischemia and life-threatening complications. A high index of suspicion, appropriate imaging, and timely surgical intervention are essential for optimal outcomes.
